# RETRACTION: A Novel Deep Learning Framework for Accurate Melanoma Diagnosis Integrating Imaging and Genomic Data for Improved Patient Outcomes

**DOI:** 10.1111/srt.70296

**Published:** 2025-11-19

**RETRACTION**: A. Kiran, N. Narayanasamy, J. V. N. Ramesh, and M. W. Ahmad, “A Novel Deep Learning Framework for Accurate Melanoma Diagnosis Integrating Imaging and Genomic Data for Improved Patient Outcomes,” *Skin Research and Technology* 30, no. 6 (2024): e13770, https://doi.org/10.1111/srt.13770.

The above article, published online on 16 June 2024 in Wiley Online Library (wileyonlinelibrary.com), has been retracted by agreement between *Skin Research and Technology*; and John Wiley & Sons Ltd. The article was accepted for publication following an insufficient peer review process that does not meet the standards of the journal's peer review policy. Furthermore, the article exhibits scientific inconsistencies between the described methodology and the reported results, includes citations that are irrelevant to the context, and lacks crucial information necessary for interpreting and reproducing the findings. Accordingly, the article has been retracted. The authors have been informed of the retraction decision but were unavailable for final confirmation.

